# Diagnostic performance of Uromonitor and TERTpm ddPCR urine tests for the non-invasive detection of bladder cancer

**DOI:** 10.1038/s41598-024-83976-2

**Published:** 2024-12-23

**Authors:** Anja Rabien, Dezhi Rong, Silke Rabenhorst, Thorsten Schlomm, Flora Labonté, Sebastian Hofbauer, Nathalie Forey, Florence Le Calvez-Kelm, Thorsten H. Ecke

**Affiliations:** 1 Department of Urology, Charité – Universitätsmedizin Berlin, corporate member of Freie Universität Berlin, Humboldt-Universität zu Berlin, and Berlin Institute of Health, Berlin, Germany; 2 Department of Urology, Helios Hospital, Bad Saarow, Germany; 3 International Agency for Research on Cancer (IARC), Lyon, France

**Keywords:** Bladder cancer, TERT mutation, Urine, Tumor marker, Non-invasive, Biomarkers, Oncology, Urology

## Abstract

Uromonitor and urinary telomerase reverse transcriptase promoter mutation droplet digital PCR (uTERTpm ddPCR) are non-invasive tests designed to detect bladder cancer in urine. We aimed to compare the diagnostic performance of uTERTpm ddPCR, Uromonitor and urine cytology in detecting bladder cancer. Urine samples were collected prospectively from patients diagnosed with primary (*n* = 74) and recurrent bladder cancer (*n* = 20) or benign urological conditions (*n* = 48) prior to surgical resection. The samples were tested for bladder cancer via uTERTpm ddPCR, Uromonitor and urine cytology. The sensitivity, specificity, and predictive values were calculated for each test, including confidence intervals. The results were stratified by low-grade non-muscle-invasive bladder cancer, high-grade non-muscle-invasive bladder cancer and muscle-invasive bladder cancer. Compared with urine cytology (59.5%, *p* = 0.005) and Uromonitor (56.8%, *p* = 0.001), the uTERTpm ddPCR test had the highest sensitivity (79.7%) for the detection of primary bladder cancer. Specificity did not significantly differ. The uTERTpm ddPCR test exhibited superior diagnostic performance over urine cytology and Uromonitor, highlighting its potential for non-invasive primary bladder cancer diagnosis.

**Supplementary Information:**

The online version contains supplementary material available at 10.1038/s41598-024-83976-2.

## Introduction

Bladder cancer (BC) ranks as the 9th most common cancer worldwide, with approximately 613,791 new cases and 220,349 deaths annually^1^. The primary diagnosis typically involves non-muscle-invasive BC (NMIBC), which is treated with transurethral resection of a bladder tumor (TURBT). NMIBC has a high recurrence and progression rate, prompting the development of various risk assessment systems^2^. Depending on the risk group classification, NMIBC requires long-term follow-up^3^. Patients with muscle-invasive BC (MIBC) face an increased risk of metastasis and require radical cystectomy, often combined with neoadjuvant chemotherapy^3^. Current diagnostic methods for BC include imaging, cystoscopy, and urine cytology as well as post-TURBT histology evaluations. While effective, cystoscopy is invasive, uncomfortable and prone to complications^4^. Urinary cytology, although highly specific, lacks sensitivity in detecting low-grade NMIBC, restricting its use as a primary BC detection method^5^. Although various urine biomarkers have been developed, their clinical validity is still being investigated^6^. Therefore, no urine biomarkers have been recommended by urological societies in routine clinical practice. The direct contact of bladder tumor cells with urine offers a unique opportunity to collect tumor-derived DNA from exfoliated tumor cells and released cell-free DNA fragments. Hotspot somatic mutations in the promoter of the telomerase reverse transcriptase gene (TERTpm) have been reported in up to 83% of cases, making them promising urinary biomarkers for BC detection^7–9^.

Droplet digital polymerase chain reaction (ddPCR) enables sensitive detection of trace amounts of DNA in a sample. A ddPCR-based assay for analyzing TERTpm in urine (uTERTpm) has been developed recently^10–12^ to detect the two most frequent C228T and C250T mutations and the rare CC242-243TT, C228A, and A161C mutations^10,11^. This assay has demonstrated high sensitivity and specificity in detecting both primary and recurrent BC^11,13^. Uromonitor, a real-time PCR assay, detects various hotspot mutations in urine, including TERTpm (C228T and C250T), fibroblast growth factor receptor 3 (FGFR3) mutations (R248C & S249C) and, more recently, Kirsten rat sarcoma oncogene viral homologue (KRAS) mutations (G12/13 & Q61)^14^. Its application as a surveillance marker for NMIBC patients has shown promising accuracy^15^. This study aimed to compare the diagnostic performance of uTERTpm ddPCR, Uromonitor and urine cytology and to investigate differences in the detection rates across BC risk subgroups.

## Materials and methods

### Study population

This case‒control study, nested within a multicenter study comparing commercially available urinary bladder cancer tests^16^, was performed at a single site and received approval from the local ethics committee of the Landesärztekammer Brandenburg (S36(bB)/2020). All participants were informed about the study’s nature and provided written informed consent. Recruitment and sample collection took place at the Department of Urology at Charité – Universitätsmedizin Berlin, Germany, from January 2021 to March 2023. Eligible participants randomly included patients referred to the department for TURBT or treatment of other urological diseases. Tissue samples from patients undergoing surgery were collected and analyzed for histological grading (1973 and 2004 World Health Organization (WHO) classification) and TNM staging. Patients with histologically confirmed BC after TURBT were categorized into the case group (94 patients), whereas those with a negative BC diagnosis after TURB (17 patients), negative cystoscopy (4 patients) or no history of BC or urologically benign cases without cystoscopy (27 patients) were assigned to the control group. The latter cases included kidney transplant donors, spermatoceles, varicoceles and others. All participants were informed about the study’s nature and provided written consent. Demographic information, smoking status, hematuria status (using a urine dipstick), and other relevant medical information were collected from all patients. The exclusion criteria included kidney stones over 3 mm, other malignant tumors, urinary tract infection, pregnancy, ongoing chemotherapy, or any mechanical manipulation of the urinary tract within the last two weeks according to the study mentioned above^16^.

## Processing of urine samples

Upon recruitment, all patients provided presurgery urine samples, which were analyzed using the urine dipstick Medi-Test URYXXON Stick 10 and the URYXXON 300 Analyzer (Macherey-Nagel, Düren, Germany) and subsequently processed within 2 h of urine retrieval. Ten milliliters of urine were sent for cytological analysis by a licensed pathologist at Charité, who assessed urine cells according to the Paris classification. Another 10 ml of urine was filtered through a 0.8 μm nitrocellulose syringe filter (Whatman^®^ Filter Z612545, Merck, Germany) with custom storage buffer provided by Uromonitor. The filter was stored at 4 °C and sent within 1 month to the Uromonitor Laboratory at UpTec (Porto, Portugal) for analysis^14^.

The remaining urine sample (20–40 ml) was allocated for TERTpm analysis following the established protocol^10^. The minimum volume of 20 ml resulted from our previous findings to obtain enough DNA in most cases, and the maximum volume was due to the limit of the DNA isolation column. The urine samples were centrifuged at 3000 × g for 15 min and divided into a urine supernatant for isolation of cell-free DNA (cfDNA) and a urine pellet for isolation of cellular DNA (cellDNA) and stored at -80 °C for up to 1 year. DNA isolation was performed using the Zymo Quick-DNA™ Urine Kit (Freiburg, Germany), and uTERTpm ddPCR analysis of the C228T, C250T, CC242-243TT, C228A, and A161C mutations was conducted with a Bio-Rad QX200™ Droplet Reader (Feldkirchen, Germany; Supplementary Table S1)^10,11^. Samples were considered positive if their respective cellDNA or cfDNA samples had at least one mutation. Details of the protocols for Uromonitor and uTERTpm ddPCR are described in the Supplementary Methods.

### Statistical analysis

For the uTERTpm ddPCR analysis, the distribution of the mutant allelic fraction (MAF) was compared via the Mann-Whitney U test (unpaired groups). The sensitivity, specificity, positive predictive value (PPV), and negative predictive value (NPV) were calculated for each test, with confidence intervals (CIs) determined via the Clopper-Pearson method. Differences in sensitivity and specificity were tested for significance using the McNemar’s chi-square test, whereas differences in PPV and NPV were tested via the relative predictive value method^17^, assuming an estimated 30% disease prevalence for symptomatic patients on the basis of Springer et al.^18^. Statistical analysis was stratified by tumor grading and calculated for total and primary BC patients. NMIBC cases were further subdivided by their risk of progression according to the European Organisation for Research and Treatment of Cancer (EORTC) criteria, based on Sylvester et al.^19^. The agreement between cfDNA and cellDNA uTERTpm status was evaluated using Wilcoxon signed-rank test (paired groups). All descriptive and inferential statistical analyses were performed via R version 4.3.1, with specific R libraries used, as shown in Supplementary Table S2. P values less than 0.05 were considered statistically significant.

All experiments were performed in accordance with relevant guidelines and regulations.

## Results

### Patient characteristics

Among the 167 patients who provided urine samples, 25 were excluded (13 had missing cytology reports, 5 had unclear cytology reports, and 7 had insufficient DNA quantity for Uromonitor/ddPCR). Ultimately, 142 patients had complete data, comprising 94 BC patients and 48 controls. Patient characteristics are provided in Table 1. Most BC cases were primary (74, 78.7%) rather than recurrent (20, 21.3%). Primary BC cases included 42 pTa cases (44.7%, 14 high-grade (HG), 28 low-grade (LG)), 17 pT1 cases (18.1%, 15 HG, 2 LG) and 15 MIBC cases with a stage of pT2 (16.0%).

**Table 1:** **Patient characteristics of the patient cohort.** BC = bladder cancer; LG/HG = low-grade/high-grade; (N)MIBC = (non-)muscle invasive bladder cancer; EORTC = European Organisation for Research and Treatment of Cancer; na = not applicable, *This includes one patient with isolated pTis without WHO histological grading assigned to it.

	BC patients (*n* = 94)	Patients with NMIBC (*n* = 73)	Patients with LG NMIBC (*n* = 39)	Patients with HG NMIBC (*n* = 34)	Patients with MIBC (*n* = 21)	Controls (*n* = 48)
**Median age**,** years (IQR)**	70 (64–78)	70 (63–78)	69 (61–76)	70 (66–79)	72 (68–77)	58 (52–67)
**Sex**,** no. (%)**						
**Female**	22 (23)	15 (21)	11 (28)	4 (12)	7 (33)	20 (42)
**Male**	72 (77)	58 (79)	28 (72)	30 (88)	14 (67)	28 (58)
**Smoking**,** no. (%)**						
**Current**	36 (38)	27 (37)	16 (41)	11 (32)	9 (43)	15 (31)
**Former**	24 (26)	21 (29)	10 (26)	11 (32)	3 (14)	9 (19)
**Never**	34 (36)	25 (34)	13 (33)	12 (35)	9 (43)	24 (50)
**Hematuria**,** no. (%)**	55 (59)	38 (52)	16 (41)	22 (65)	17 (81)	8 (17)
**Missing**	-	-	-	-	-	5 (10)
**Primary tumor**,** no. (%)**	74 (79)	59 (81)	30 (77)	29 (85)	15 (71)	48 (100)
**TNM**,** no. (%)**						
**pTa**	53 (56)	53 (73)	37 (95)	16 (47)	0 (0)	-
**-pTa with carcinoma in situ (%)**	4 (4)	4 (5)	0 (0)	4 (12)	0 (0)	
**pTis**	1 (1)	1 (1)	0 (0)	1 (3)	0 (0)	-
**pT1**	19 (20)	19 (26)	2 (5)	17 (50)	0 (0)	-
**-pT1 with carcinoma in situ (%)**	3 (3)	3 (4)	0 (0)	3 (9)	0 (0)	
**pT2**	20 (21)	0 (0)	0 (0)	0 (0)	20 (95)	-
**-pT2 with carcinoma in situ (%)**	1 (1)	0 (0)	0 (0)	0 (0)	1 (5)	
**pT3**	0 (0)	0 (0)	0 (0)	0 (0)	0 (0)	-
**pT4**	1 (1)	0 (0)	0 (0)	0 (0)	1 (5)	-
**Carcinoma in situ**,** no. (%)**	9 (10)	8 (11)	0 (0)	8 (24)	1 (4.8)	-
**Histological grading (WHO 1973)**,** no. (%)**						
**G1**	15 (16)	15 (21)	15 (38)	0 (0)	0 (0)	-
**G2**	35 (37)	33 (45)	24 (62)	9 (26)	2 (10)	-
**G3**	43 (46)	24 (33)	0 (0)	24 (71)	19 (90)	-
Not available*	1 (1)	0 (0)	0 (0)	1 (3)	0 (0)	
**Number of tumors**,** no. (%)**						
**Solitary**		41 (56)	25 (64)	16 (47)	n.a.	-
**Multiple**		32 (44)	14 (36)	18 (53)	n.a.	-
**Maximum diameter**,** no. (%)**						
**<3 cm**		47 (64)	28 (72)	19 (56)	n.a.	-
**≥3 cm**		26 (36)	11 (28)	15 (44)	n.a.	-
**EORTC risk of progression**,** no. (%)**						
**Low risk**	-	15 (21)	14 (36)	1 (3)	-	-
**Intermediate risk**	-	33 (45)	24 (62)	9 (26)	-	-
**High risk**	-	25 (34)	1 (3)	24 (71)	-	-

Table 1.

### Description of the urine test results

**Fig. 1:**
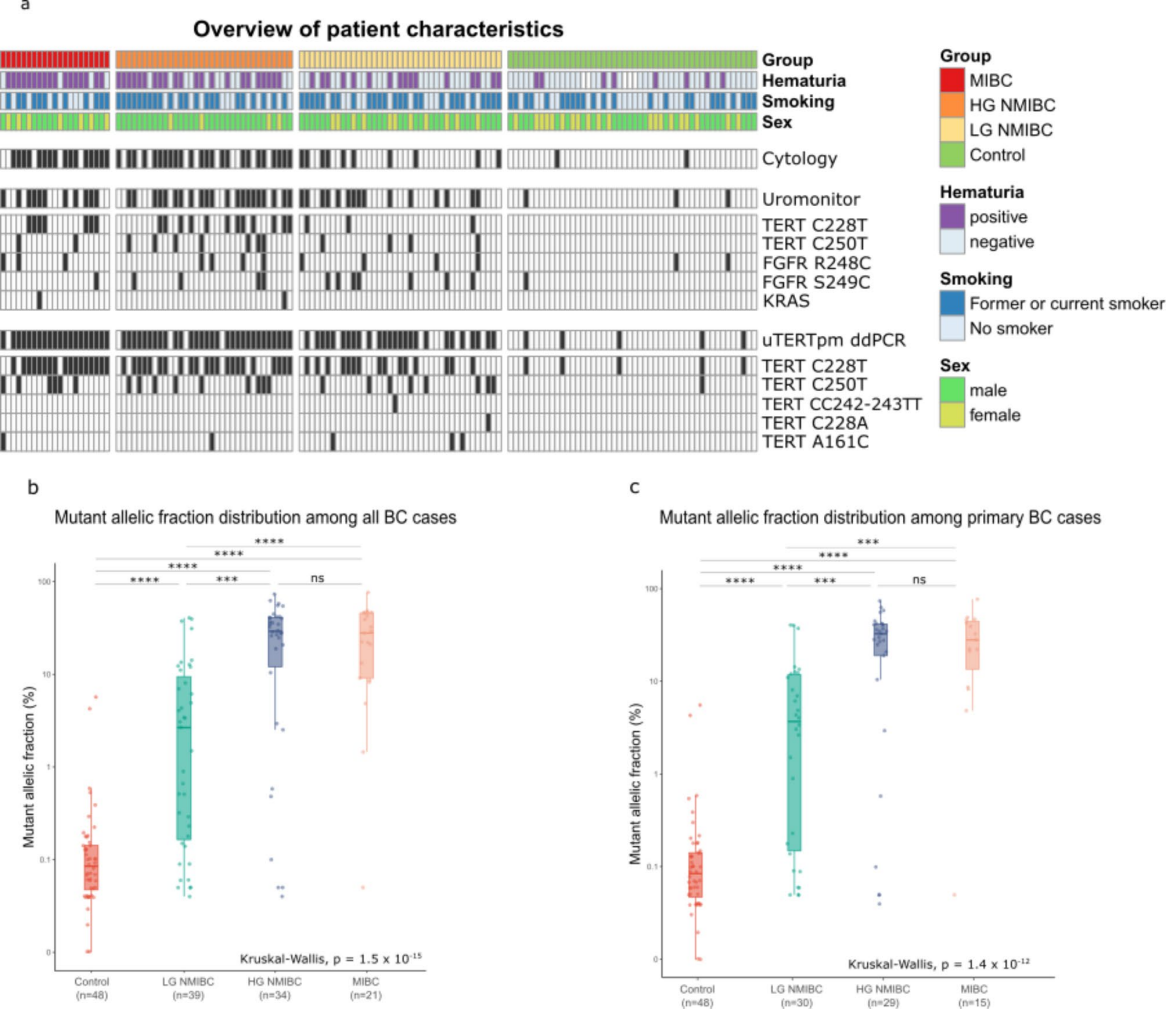
**Results of urine cytology**,** Uromonitor and uTERTpm ddPCR.** (a) Heatmap overview of patient characteristics. The groups were stratified into LG NMIBC, HG NMIBC, MIBC, and control groups. Information on gross hematuria, smoking, and sex was collected at recruitment. An overview of the test results for the cytology, Uromonitor and uTERTpm ddPCR results is shown below, with black bars denoting positive test results. b, c) Boxplot and dots displaying the distribution of mutant allelic fractions from uTERTpm ddPCR, (b) in all BC cases and (c) in primary BC cases. For each sample, an aggregate was built by picking the highest measurement from all five mutations. The boxplot denotes the median, 1st and 3rd quartiles, and the whiskers denote the 1.5 interquartile range. Individual samples are represented by dots. Differences in medians were tested with the Mann-Whitney U test. All p values < 0.05 were considered significant; otherwise, they were considered not significant (ns). ** *p* < 0.01, *** *p* < 0.001, **** *p* < 0.0001. BC = bladder cancer, LG/HG = low-grade/high-grade, (N)MIBC = (non-)muscle invasive bladder cancer.

Among the 94 confirmed BC patients, 76 had positive uTERTpm results (mutation(s) detected in urine cfDNA or cell DNA), 50 were positive for cytology, and 47 had positive Uromonitor results (Fig. 1a, Supplementary Table S3. Both the Uromonitor and the uTERTpm ddPCR tests were positive for 43 BC patients and negative for 14 BC patients. The uTERTpm ddPCR assay identified 33 cases negative by Uromonitor (30 with C228T or C250T mutations targeted by Uromonitor), whereas Uromonitor identified 4 uTERTpm-negative BC patients (all positive for FGFR3 mutations, not targeted by ddPCR). There were 5 and 3 false positives with uTERTpm ddPCR and Uromonitor, respectively. The most common mutations were C228T and C250T, followed by A161C, C228A, and CC242-243TT. Only three BC patients had rare uTERTpm ddPCR mutations without a concomitant C228T or C250T mutation. Uromonitor revealed 27 patients with FGFR3 mutations and two with KRAS mutations, with important overlap between FGFR3 mutations (15/27) or KRAS (2/2) and TERTpm, so that KRAS mutations did not show any benefit in our study (Supplementary Table S3).

The uTERTpm ddPCR mutational fractions (MAFs) were associated with the tumor histological subgroups. Patients with LG NMIBC had a significantly lower MAF (median 2.66%) than did those with HG NMIBC (median 29.18%) or MIBC (median 27.88%) (Fig. 1b). Similar results were observed for primary BC only (Fig. 1c). The controls had the lowest median MAF, with the majority below the range of the limit of detection, which was previously determined^11^ and validated again in this study for C228T (Supplementary Table S4). An overview of the MAFs across all 5 mutations is given in Supplementary Table S5. Of the 142 cases tested for uTERTpm, 102 yielded uTERTpm results of both, cellular DNA and cfDNA, among them 73 BC patients and 29 controls (Supplementary Fig. S1). Especially for controls and their cfDNA, the amount of DNA was often under the limit to be included in the analysis so that the number of matched DNA results was low. Nevertheless, the 102 matched cases in total revealed that the MAFs for both DNAs were mostly similar or determined by higher MAF of cellular DNA (higher MAF was used for calculation) so that division will not be necessary for experiments in the future. Native urine will be sufficient for DNA isolation.

While the uTERTpm clearly outperformed the other tests for LG NMIBC, combined tests increased the detection rate of HG NMIBC. Compared with uTERTpm alone (30/34, 88.2%) and cytology alone (24/34, 70.6%), uTERTpm ddPCR together with urine cytology detected a higher proportion of 33/34 (97.1%) HG NMIBC cases. Compared with Uromonitor alone (21/34, 61.8%) or cytology alone (24/34, 70.6%), Uromonitor combined with cytology also increased the detection rate of HG NMIBC, with 26/34 (76.5%). However, Uromonitor was not able to improve the uTERTpm detection rate of 88.2%.

## Sensitivities and specificities of urine tests

**Fig. 2:**
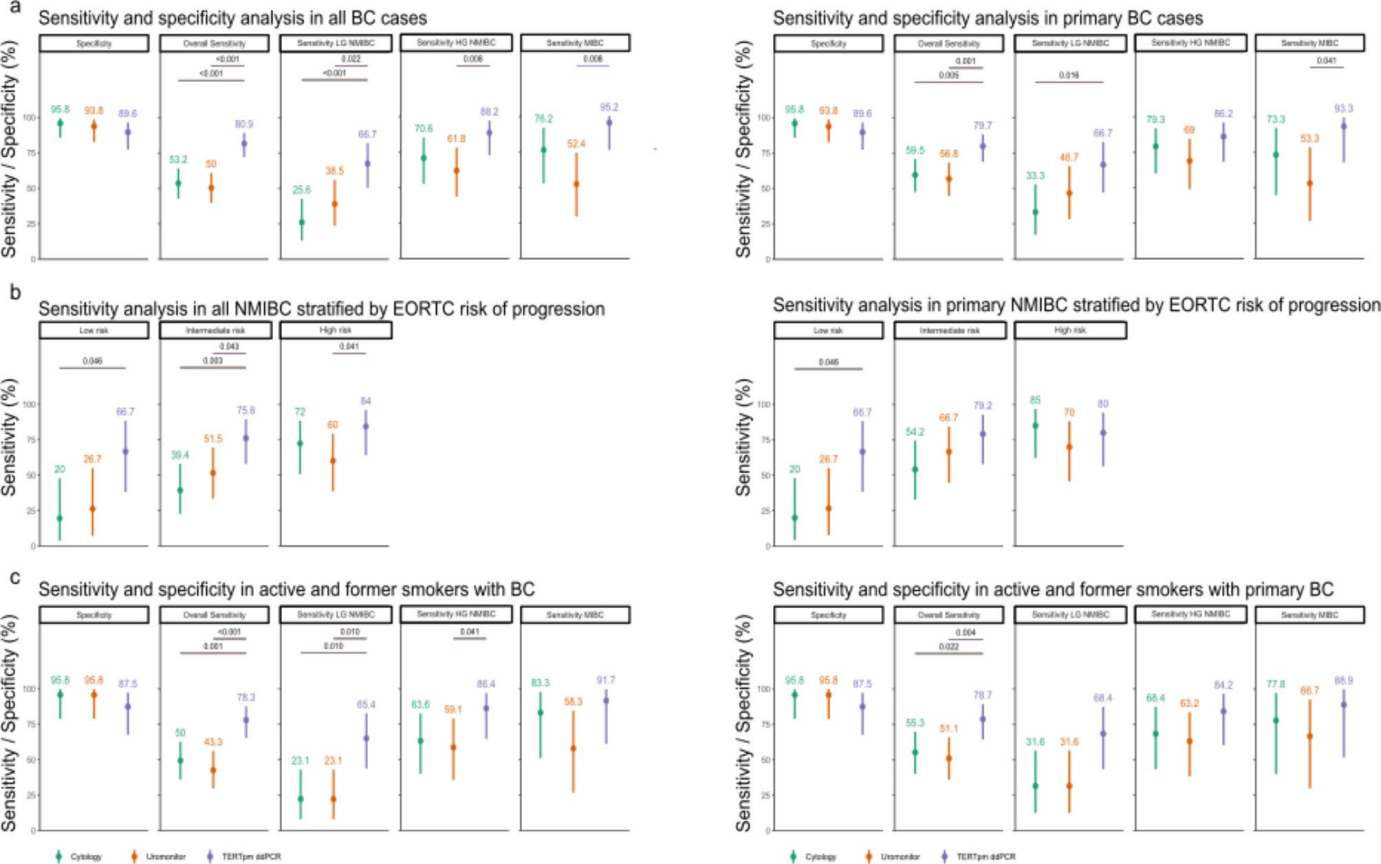
**Overall and stratified sensitivities and specificities of urine cytology**,** Uromonitor and uTERTpm ddPCR tests in detecting BC.** Specificity against controls (*n* = 48) and sensitivity are displayed (a) for all BC patients (*n* = 94, left) and for primary BC patients (*n* = 74, right). (b) NMIBC stratified according to EORTC risk scores are shown for all BC cases (left) and for primary BC cases (right). (c) Stratification according to active and former smoker status is given for all BC cases (left) and for primary BC cases (right). Significance was calculated with the McNemar chi square test. All p values < 0.05 were considered significant. ** *p* < 0.01, *** *p* < 0.001, **** *p* < 0.0001. uTERTpm ddPCR = urinary telomerase reverse transcriptase promoter mutation droplet digital polymerase chain reaction; BC = bladder cancer; LG/HG = low-grade/high-grade; (N)MIBC = (non-)muscle invasive bladder cancer; EORTC = European Organisation for Research and Treatment of Cancer.

uTERTpm ddPCR showed higher sensitivity for detecting all (80.9%; 95% CI 71.4–88.2) and primary (79.7%; 95% CI 68.8–88.2) BC cases than did urine cytology (53.2%; 95% CI 42.6–63.6; *p* < 0.0001 and 59.5%; CI 47.7–70.7; *p* = 0.0051) and Uromonitor (50.0%; 95% CI 39.5–60.5; *p* < 0.0001 and 56.8%, 95% CI 44.7–68.2; *p* = 0.001) (Table 2; Fig. 2a). Urine cytology showed the highest specificity (95.8%, 95% CI 85.7–99.5) but the differences were not significant. Stratified analysis revealed superior sensitivity for uTERTpm ddPCR across BC subgroups compared with Uromonitor, including LG NMIBC (66.7% vs. 38.5%; *p* = 0.022), HG NMIBC (88.2% vs. 61.8%; *p* = 0.008) and MIBC (95.2% vs. 52.4%; *p* = 0.008). It also outperformed cytology for LG NMIBC (66.7% vs. 25.6%; *p* < 0.001). For primary BC, uTERTpm ddPCR exhibited higher sensitivity than did urine cytology for LG NMIBC (66.7%; 95% CI 47.2–82.7 versus 33.3%; 95% CI 17.3–52.8; *p* = 0.016) and Uromonitor for MIBC (93.3% vs. 53.3%; *p* = 0.041) (Table 2; Fig. 2a right). When NMIBC cases were stratified by their EORTC risk of progression, uTERTpm ddPCR was superior in detecting patients at all risk levels (Fig. 2b left). Primary BC patients with low- or intermediate-risk were most likely to be detected by uTERTpm ddPCR; however, urine cytology displayed slightly higher sensitivity for EORTC high-risk NMIBC patients than did uTERTpm ddPCR (Fig. 2b right). Interestingly, when stratifying for active and former smokers across all BC and primary cases, uTERTpm ddPCR maintained high sensitivity and specificity for LG NMIBC as compared to cytology and Uromonitor (Fig. 2c).

**Table 2:** Diagnostic performance of urine cytology, Uromonitor, and uTERTpm ddPCR in the whole BC cohort (n = 94) and in primary BC (*n* = 74). PPV and NPV were calculated using an estimated prevalence of 30% according to Springer et al. [18]. BC = bladder cancer; PPV/NPV = positive/negative predictive value; uTERTpm ddPCR = urinary telomerase reverse transcriptase promoter mutation droplet digital polymerase chain reaction; LG/HG = low-grade/high-grade; (N)MIBC = (non-)muscle invasive bladder cancer.

		All BC + control (*n* = 142)				Primary BC + control (*n* = 122)		
		Cytology	Uromonitor	TERTpm ddPCR		Cytology	Uromonitor	TERTpm ddPCR
	**False positives**,** no**	2	3	5		2	3	5
	**True negatives**,** no**	46	45	43		46	45	43
	**Specificity (%**,** 95% CI)**	95.8 (85.7–99.5)	93.8 (82.8–98.7)	89.6 (77.3–96.5)		95.8 (85.7–99.5)	93.8 (82.8–98.7)	89.6 (77.3–96.5)
**All grades**	**True positives**,** no**	50	47	76		44	42	59
	**False negatives**,** no**	44	47	18		30	32	15
	**Sensitivity (%**,** 95% CI)**	53.2 (42.6–63.6)	50.0 (39.5–60.5)	80.9 (71.4–88.2)		59.5 (47.4–70.7)	56.8 (44.7–68.2)	79.7 (68.8–88.2)
	**PPV (%**,** 95% CI)**	84.6 (58.2–95.6)	77.4 (53.0–91.3)	76.9 (59.0–88.5)		86.0 (60.9–96.0)	79.6 (56.1–92.2)	76.6 (58.7–88.3)
	**NPV (%**,** 95% CI)**	82.7 (79.3–85.7)	81.4 (77.9–84.4)	91.6 (87.7–94.4)		84.7 (80.6–88.0)	83.5 (79.4–86.9)	91.2 (86.7–94.2)
**LG NMIBC**	**True positives**,** no**	10	15	26		10	14	20
	**False negatives**,** no**	29	24	13		20	16	10
	**Sensitivity (%**,** 95% CI)**	25.6 (13.0–42.1)	38.5 (23.4–55.4)	66.7 (49.8–80.9)		33.3 (17.3–52.8)	46.7 (28.3–65.7)	66.7 (47.2–82.7)
	**PPV (%**,** 95% CI)**	72.5 (38.0–91.9)	72.5 (45.1–89.4)	73.3 (53.8–86.6)		77.4 (44.6–93.6)	76.2 (50.1–91.1)	73.3 (53.5–86.7)
	**NPV (%**,** 95% CI)**	75.0 (71.3–78.5)	78.0 (73.3–82.2)	86.3 (79.9–90.8)		77.0 (72.1–81.3)	80.4 (74.4–85.3)	86.3 (78.9–91.3)
**HG NMIBC**	**True positives**,** no**	24	21	30		23	20	25
	**False negatives**,** no**	10	13	4		6	9	4
	**Sensitivity (%**,** 95% CI)**	70.6 (52.5–84.9)	61.8 (43.6–77.8)	88.2 (72.5–96.7)		79.3 (60.3–92.0)	69.0 (49.2–84.7)	86.2 (68.3–96.1)
	**PPV (%**,** 95% CI)**	87.9 (64.8–96.6)	80.9 (57.8–92.9)	78.4 (61.1–89.4)		89.1 (67.5–97.0)	82.6 (60.6–93.6)	78.0 (60.4–89.2)
	**NPV (%**,** 95% CI)**	88.4 (81.8–92.8)	85.1 (78.8–89.8)	94.7 (87.6–97.8)		91.5 (84.1–95.7)	87.6 (80.3 – 92.4)	93.8 (85.9–97.4)
**MIBC**	**True positives**,** no**	16	11	20		11	8	14
	**False negatives**,** no**	5	10	1		4	7	1
	**Sensitivity (%**,** 95% CI)**	76.2 (52.8–91.8)	52.4 (29.8–74.3)	95.2 (76.2–99.9)		73.3 (44.9–92.2)	53.3 (26.6–78.7)	93.3 (68.1–99.8)
	**PPV (%**,** 95% CI)**	88.7 (66.4–96.9)	78.2 (52.7–92.0)	79.7 (63.0–90.0)		88.3 (65.3–96.8)	78.5 (52.6–92.4)	79.3 (62.4–89.9)
	**NPV (%**,** 95% CI)**	90.4 (81.3–95.3)	82.1 (74.5–87.9)	97.8 (86.6–99.7)		89.4 (78.3–95.1)	82.4 (73.1–89.0)	96.9 (82.5–99.5)

Table 2.

## Discussion

This study is the first to compare the diagnostic performance of uTERTpm ddPCR, Uromonitor and urine cytology for the non-invasive detection of BC. Our results indicate that uTERTpm ddPCR has by far the highest detection rate, which is consistent with previous studies reporting 67.7-86.8% sensitivity and 88-100% specificity in French, Portuguese and Iranian case-control series^11,13^. uTERTpm ddPCR yielded the highest sensitivity across all BC subgroups, as shown previously with different uTERTpm assays^13,20,21^. We confirmed the significant diagnostic value of the uTERTpm ddPCR, particularly in patients with LG NMIBC, for whom cytology has poor sensitivity^5,20^. For HG NMIBC, we showed that combining uTERTpm ddPCR with cytology could detect almost all cases, considerably improving the detection rate of BC as compared to both independent tests.

The performance of Uromonitor for the primary detection of BC and surveillance of NMIBC was first described in 2019^14^. Uromonitor detected 56.8% of all primary BC cases in our study, which is slightly higher than its previously reported sensitivity (50%, without considering KRAS mutations)^14^. However, testing for KRAS mutations with Uromonitor did not improve sensitivity in our study, as all the KRAS-positive patients carried at least one other mutation. Uromonitor did not outperform urine cytology, neither in our present work nor in the recent multicenter study we were part of^22^. Despite its limited sensitivity for primary BC, cohort studies focusing on Uromonitor’s assessment of NMIBC follow-up have reported much higher sensitivities between 73.5% and 93.1%^14,15,23,24^, highlighting its suitability for BC surveillance. Notably, ddPCR detected 30 more uTERTpm C228T or C250T cases than Uromonitor did, 19/30 with a MAF < 5% but also 9/30 with a MAF > 20%. Additionally, approximately 83% of the samples detected by Uromonitor had a uTERTpm C228T or C250T MAF > 20%. This highlights the technical superiority of uTERTpm ddPCR analysis in detecting low allelic fractions but also high allelic fractions, although it is a single-gene assay. The lower performance of Uromonitor could be partly explained by the smaller volume of urine used for the test (10 ml), which could lead to an underrepresentation of low-level mutated DNA that may be missed by Uromonitor. However, Uromonitor also failed to detect high allelic fraction mutations, which cannot be explained by inadequate urine volume and low DNA yield. Optimization concerning pre-analytical steps, i.e. filtration and DNA extraction could be envisaged although inherent technical inferiority of qPCR over ddPCR in detecting mutation would remain.

Evidence suggests that TERT promoter mutations are detectable in urine for up to 10 years before diagnosis^25^, making it a promising early detection biomarker. However, untargeted screening may not be cost effective. A study, in which asymptomatic patients were screened via urine dipsticks and molecular testing, resulted in a low diagnostic yield compared with its cost (5 tumors detected in 1747 men) but reduced unnecessary cystoscopies from 368 to 66^26^. Targeted screening of high-risk populations, e.g., smokers or those exposed to bladder carcinogens, might be a more efficient alternative. Despite the low number of cases in the subgroup, our exploratory analysis suggested that uTERTpm ddPCR retained high diagnostic performance in smokers and ex-smokers, assuming that it may have potential in screening this population. Further studies with larger cohorts and longitudinal observations are needed to fully assess the viability of uTERTpm ddPCR in BC screening.

We used strict exclusion criteria chosen according to our previous study^16^ to avoid false-positive cytology results due to other diseases, e.g., infections, but subsequent studies should include a broader spectrum of patients to better assess screening possibilities of the uTERTpm assay. Nevertheless, our urine cytology results, assessed by routine pathology, may have considerable bias because the percentages of atypical urothelial cells were high: 47.3% of primary BC patients and 25.0% of recurrent BC patients. According to the most recent expert´s opinion, the percentage of urine samples with atypical urothelial cells should not exceed 15%, as recommended in a Springer book on the Paris classification^27^. Different methods exist to classify urine cytology samples with atypical urothelial cells. We classified the results as follows: Atypical urothelial cells of primary BC were considered positive for BC, since cases with atypical cells have a remarkable percentage of high-grade BC^27^. Recurrent cases with atypical urothelial cells in cytology at detection of recurrence and six weeks or more after therapies such as BCG were also assumed to be positive since post-TURBT residual cells of the preceding cancer should then be eliminated^27^. In our panel all cases met the criteria to be marked as positive. Differences between studies examining urine cytology probably originate from different pathological assessments and the following classifications.

In this case-control study, we investigated the potential of two tests utilizing the uTERTpm ddPCR as a biomarker for primary BC detection, although other tests exist. UroMuTERT, a single-plex sensitive NGS-based assay^20^, demonstrated high specificity (93.6% and 98%) and sensitivity (86.7% and 68%) in French and Portuguese cohorts. UroMuTERT and uTERTpm ddPCR subsequently showed comparable detection rates^11^. UroSeek^18^, another assay, includes TERTpm analysis, aneuploidy, and genetic mutations in ten additional genes. While UroSeek reports slightly higher sensitivity (83% vs. 79.7%) and specificity (93% vs. 89.6%) than does TERTpm ddPCR, the simplicity of TERTpm ddPCR makes it more suitable for routine diagnosis. Furthermore, UroSeek detected TERTpm in only 57% of the cases, indicating that TERTpm ddPCR is superior in identifying TERTpm in urine.

Current research on urinary biomarkers has focused primarily on their role in BC detection, independent of cancer risk categories. Previous studies have suggested the use of tumor-derived DNA levels to discriminate low-risk patients from high-risk patients^28^, offering a significant asset in BC management. In our current study, we found a significant association between tumor grading and the uTERTpm MAF. Importantly, the uTERTpm MAF was associated with EORTC risk groups for tumor progression in NMIBC patients, specifically distinguishing between low/intermediate- and high-risk NMIBC patients. These results, though to be treated with caution due to low sample numbers, are consistent with previous research, although a different risk scoring system was used^20^. While estimating the risk of progression in BC typically relies, among other factors, on pathological staging and grading^3^, which cannot be performed immediately after BC diagnosis, uTERTpm MAF levels could provide critical clinical information on tumor growth and progression before surgical intervention, facilitating timely clinical decision-making. Limitations of our study include limited urine volumes and different urine volumes provided between subjects that resulted in various urine volume input for TERTpm analysis by ddPCR. However, it has been reported that there is considerable between-individual and disease-status variability in terms of urinary DNA yield, which complicates strict standardization of the urine DNA-based analysis. Compared with healthy individuals, the urine of whom may yield very low amounts of DNA, patients with bladder cancer often have more DNA in their urine. Standardization of a minimum amount of DNA for urine tests could be recommended but is sometimes hard to achieve for healthy controls. Moreover, patients with benign urological diseases do not undergo supplementary examinations beyond standard management. Missing information on the upper urinary tract in the enrolled controls could have potentially contributed to “false-positive” urine test results in undiagnosed patients with carcinoma of the upper urinary tract. We collected as many cases as possible in the (pandemic) project time, and the sample size, however, hampered statistical power when stratifying BC subgroups. A larger multicenter screening study should follow our proof-of-concept work, which was limited by the strict exclusion criteria applied^16^.

However, despite these limitations, we could show that the uTERTpm ddPCR test outperformed urine cytology and Uromonitor in detecting primary and overall BC. Its improved detection rate, especially for LG NMIBC, makes it a promising tool for early-stage BC diagnosis and potential non-invasive screening in high-risk populations. Additionally, uTERTpm allelic fraction levels are suggested to strongly facilitate the detection of HG NMIBC, supporting cytology. Large longitudinal studies are needed to fully assess the clinical importance of uTERTpm ddPCR for BC diagnosis and screening.

## Electronic supplementary material

Below is the link to the electronic supplementary material.

Supplementary Material 1

## Data Availability

Dezhi Rong has full access to all the data of the study and takes responsibility for the integrity of the data and the accuracy of the data analysis. The raw data can be obtained upon request.
