# Supplementary material for: Diagnostic performance of Uromonitor and TERTpm ddPCR urine tests for the non-invasive detection of bladder cancer
Source: Sci Rep. 2024 Dec 23;14:30617. doi: 10.1038/s41598-024-83976-2 (PMC11666540; doi:10.1038/s41598-024-83976-2)
Supplement: Supplementary file 1 — Supplementary Material 1 [file 41598_2024_83976_MOESM1_ESM.docx]

**Supplementary information**

**Supplementary Table S1 – Digital droplet PCR protocol information**

| **Mutation** | **Amplification protocol**  10min 95°C  then 40 custom cycles (see below)  then 10min 98°C  then 30min 4°C | **Amplitude threshold Mut** | **Amplitude threshold WT** | **Minimum number of positive droplets for calling a mutation** |
| --- | --- | --- | --- | --- |
| C228T | 30s 94°C denaturation  30s 54°C annealing | 1300 | 1800 | 6 |
| C250T | 30s 94°C denaturation  30s 64°C annealing | 3000 | 2000 | 5 |
| CC242-243TT | 30s 94°C denaturation  30s 55°C annealing | 1000 | 1600 | 6 |
| C228A | 30s 94°C denaturation  30s 55°C annealing | 830 | 1550 | 5 |
| A161C | 30s 96°C denaturation (50 cycles)  60s 62°C annealing (50 cycles) | 2500 | 2000 | 3 |

**Supplementary methods**

Uromonitor

As described by Batista et al. 2019, the Uromonitor laboratory uses real-time PCR for TERTpm amplification and analysis. For TERTp C228T and C250T mutations, specific Locked Nucleic Acid probes had been developed for their respective real-time allelic discrimination assays. For FGFR3 and KRAS mutations, a competitive allele-specific assay had been designed with specific mutation allele-specific primers and phosphorylated wild-type allele blockers to suppress the amplification of the wild-type allele. The analytical limit of detection was 6.25% mutant allelic fraction, calling mutations above this detection limit.

Batista R, Vinagre J, Prazeres H et al. Validation of a Novel, Sensitive, and Specific Urine-Based Test for Recurrence Surveillance of Patients With Non-Muscle-Invasive Bladder Cancer in a Comprehensive Multicenter Study. Frontiers in Genetics 2019; 10:1237.

uTERTpm ddPCR

After DNA isolation from urine cell pellet or supernatant, DNA was quantified using QubitTM 1X dsDNA BR Assay Kit and QubitTM 1X dsDNA HS Assay Kit on the QubitTM® 4 Fluorometer (Invitrogen, Thermo Fisher, Waltham, MA, USA).

For each ddPCR measurement, we used a 22µl reaction mix consisting of 10ng DNA (or 5ng DNA in case of low amounts), 11µl of 2x ddPCR supermix-no dUTP (Biorad), 1.1µl 20xFAM and 1.1µl 20xHEX probes for mutated and wild type alleles, 1.1µl Rsal restriction enzyme (10U/µl) and 0.2µl of 7-deaza-dGTP, Li-salt. Droplets were generated manually using the Bio-Rad QX200™ Droplet Generator. Droplet amplification was performed using Bio-Rad T100 Thermal Cycler (amplification protocols provided in Supplementary Table 1). Droplet Fluorescence was measured with a Bio-Rad QX200™ Droplet Reader.

The fluorescence data and 2D-amplitude plots were analyzed using the QuantaSoftTM Analysis Pro 1.7.4.0917 software (Biorad). Threshold amplitudes for the mutated and wild-type channels were set at 1300/1800 (C228T), 3000/2000 (C250T), 1000/1600 (CC242-243TT), 830/1550 (C228A), 2500/2000 (A161C) respectively. Only measurements with a minimum number of 10.000 accepted droplets and 500 positive droplets were considered for mutational analysis.

The threshold for the minimum number of positive droplets for calling a mutation was set at 6 (C228T/CC242-243TT assays), 5 (C228A/C250T assays), and 3 (A161C assay) according to previous results (Hosen et al., 2020) and our confirmation for C228T (Supplementary Table 3). Results from cellDNA and cfDNA analysis were combined by aggregating the highest MAF to create a whole urine result.

Hosen MI, Forey N, Durand G et al. Development of Sensitive Droplet Digital PCR Assays for Detecting Urinary TERT Promoter Mutations as Non-Invasive Biomarkers for Detection of Urothelial Cancer. Cancers 2020; 12 (12).

**Supplementary Table S2 – R libraries used for data analysis**

| dcurves | Decision curve analysis |
| --- | --- |
| epiR | Sensitivity, specificity, predictive values, incl. 95% confidence intervals |
| stats | Comparison between sensitivity and specificity |
| DTComPair | Comparison between predictive values |

**Supplementary Table S3 - Test results for urine cytology, Uromonitor and TERTpm ddPCR.**

BC = bladder cancer; LG/HG = low-grade/high-grade; (N)MIBC = (non-)muscle invasive bladder cancer; TERTpm ddPCR = telomerase reverse transcriptase promoter mutation droplet digital polymerase chain reaction.

|  | **BC patients**  **(n = 94)** | **BC patients with NMIBC (n=73)** | **BC patients with LG NMIBC**  **(n = 39)** | **BC patients with HG NMIBC**  **(n = 34)** | **BC patients with MIBC**  **(n = 21)** | **Controls**  **(n = 48)** |
| --- | --- | --- | --- | --- | --- | --- |
| **Urine cytology**  **no. (%)** |  |  |  |  |  |  |
| **Positive** | 50 (53) | 34 (47) | 10 (26) | 24 (71) | 16 (76) | 2 (4) |
| **Negative** | 44 (47) | 39 (53) | 29 (74) | 10 (29) | 5 (24) | 46 (96) |
| **Uromonitor, no. (%)** |  |  |  |  |  |  |
| **Positive** | 47 (50) | 36 (49) | 15 (38) | 21 (62) | 11 (52) | 3 (6) |
| **uTERTpm** |  |  |  |  |  |  |
| **C228T** | 16 (17) | 11 (15) | 2 (5) | 9 (26) | 5 (24) | 0 (0) |
| **C250T** | 7 (7) | 6 (8) | 2 (5) | 4 (12) | 1 (5) | 0 (0) |
| **FGFR3** |  |  |  |  |  |  |
| **R248C** | 6 (6) | 4 (5) | 3 (8) | 1 (3) | 2 (10) | 2 (4) |
| **S249C** | 6 (6) | 6 (8) | 5 (13) | 1 (3) | 0 (0) | 1 (2) |
| **Double/triple mutations** |  |  |  |  |  |  |
| **C228T and R248C** | 1 (1) | 1 (1) | 0 (0) | 1 (3) | 0 (0) | 0 (0) |
| **C228T and S249C** | 3 (3) | 2 (3) | 1 (3) | 1 (3) | 1 (5) | 0 (0) |
| **C228T and KRAS G12/13** | 2 (2) | 1 (1) | 0 (0) | 1 (3) | 1 (5) | 0 (0) |
| **C250T and R248C** | 3 (3) | 2 (3) | 1 (3) | 1 (3) | 1 (5) | 0 (0) |
| **C250T and S249C** | 2 (2) | 2 (3) | 1 (3) | 1 (3) | 0 (0) | 0 (0) |
| **C250T and R248C and S249C** | 1 (1) | 1 (1) | 0 (0) | 1 (3) | 0 (0) | 0 (0) |
|  |  |  |  |  |  |  |
| **Negative** | 47 (50) | 37 (51) | 24 (62) | 13 (38) | 10 (48) | 45 (94) |
| **uTERTpm ddPCR**  **no. (%)** |  |  |  |  |  |  |
| **Positive** | 76 (81) | 56 (77) | 26 (67) | 30 (88) | 20 (95) | 5 (10) |
| **C228T** | 48 (51) | 34 (47) | 14 (36) | 20 (59) | 14 (67) | 4 (8) |
| **C250T** | 17 (18) | 15 (21) | 7 (18) | 8 (24) | 2 (10) | 0 (0) |
| **CC242-243TT** | 1 (1) | 1 (1) | 1 (3) | 0 (0) | 0 (0) | 0 (0) |
| **C228A** | 0 (0) | 0 (0) | 0 (0) | 0 (0) | 0 (0) | 0 (0) |
| **A161C** | 2 (2) | 2 (3) | 2 (5) | 0 (0) | 0 (0) | 0 (0) |
| **Double mutations** |  |  |  |  |  |  |
| **C228T and C250T** | 4 (4) | 1 (1) | 0 (0) | 1 (3) | 3 (15) | 1 (2) |
| **C228T and C228A** | 0 (0) | 0 (0) | 0 (0) | 0 (0) | 0 (0) | 0 (0) |
| **C228T and A161C** | 2 (2) | 2 (3) | 1 (3) | 1 (3) | 0 (0) | 0 (0) |
| **C250T and C228A** | 1 (1) | 1 (1) | 1 (3) | 0 (0) | 0 (0) | 0 (0) |
| **C250T and A161C** | 1 (1) | 0 (0) | 0 (0) | 0 (0) | 1 (5) | 0 (0) |
| **Negative** | 18 (19) | 17 (23) | 13 (33) | 4 (12) | 1 (5) | 43 (90) |

**Supplementary Table S4: Limit of detection for C228T mutations for 5ng and 10ng input DNA**

| Sample | Total Number of DNA droplets | Number of mutated DNA droplets | Mutant Allelic Fraction |
| --- | --- | --- | --- |
| HT1376 5ng (100% mutation) | 1497 | 487 | 32.532 |
| HT1376 5ng dilut. 1:2 | 1330 | 205 | 15.414 |
| HT1376 5ng dilut. 1:5 | 1386 | 85 | 6.133 |
| HT1376 5ng dilut. 1:10 | 1415 | 46 | 3.251 |
| HT1376 5ng dilut. 1:20 | 1545 | 31 | 2.006 |
| HT1376 5ng dilut. 1:50 | 1545 | 14 | 0.906 |
| HT1376 5ng dilut. 1:100 | **1415** | **7** | **0.495** |
| HT1376 5ng dilut. 1:200 | 1550 | 1 | 0.065 |
| HT1376 5ng dilut. 1:500 | 1514 | 0 | 0.000 |
| HT1376 5ng dilut. 1:1000 | 1497 | 3 | 0.200 |
| Caki1 5ng (100% wild type) | 1535 | 1 | 0.065 |
| HT1376 10ng (100% mutation) | 3424 | 1.130 | 33.002 |
| HT1376 10ng dilut. 1:2 | 2978 | 460 | 15.447 |
| HT1376 10ng dilut. 1:5 | 2859 | 172 | 6.016 |
| HT1376 10ng dilut. 1:10 | 2516 | 79 | 3.140 |
| HT1376 10ng dilut. 1:20 | 2500 | 52 | 2.080 |
| HT1376 10ng dilut. 1:50 | 2627 | 24 | 0.914 |
| HT1376 10ng dilut. 1:100 | 2482 | 8 | 0.322 |
| HT1376 10ng dilut. 1:200 | **2859** | **6** | **0.210** |
| HT1376 10ng dilut. 1:500 | 2491 | 3 | 0.120 |
| HT1376 10ng dilut. 1:1000 | 2707 | 1 | 0.037 |
| Caki1 10ng (100% wild type) | 2785 | 1 | 0.036 |
| Non-template control | 1 | 0 | 0.000 |

**Supplementary Table S5: List of Uromonitor and uTERTpm results related to group, tumor history, and Mutant Allelic Fraction (MAF)**

| Group | Tumor history | Uromonitor | uTERTpm | MAF Cell.  C228T | MAF  Cell Free C228T | MAF Cell.  C250T | MAF  Cell Free  C250T | MAF Cell.  C242T | MAF  Cell Free  C242T | MAF Cellular  C228A | MAF  Cell Free  C228A | MAF Cell.  A161C | MAF  Cell Free  A161C |
| --- | --- | --- | --- | --- | --- | --- | --- | --- | --- | --- | --- | --- | --- |
| HGNMIBC | No | negative | negative | 0 | 0,05 | 0,07 | 0,1 | 0 | 0 | 0 | 0 | 0 | NA |
| LGNMIBC | No | c.742C>T FGFR3 | negative | 0,05 | 0,05 | 0,05 | 0,23 | 0 | 0 | 0 | 0 | 0 | NA |
| LGNMIBC | No | c.-124C>T TERT | C228T | 13,56 | NA | 0 | NA | 0 | NA | 0 | NA | 0 | NA |
| HGNMIBC | No | negative | C228T | 36,72 | 16,6 | 0,05 | 0 | 0 | 0 | 0 | 0 | 0 | 0 |
| Control | No | negative | negative | 0,13 | 0,03 | 0,04 | 0 | 0 | 0 | 0 | 0 | 0 | 0 |
| HGNMIBC | No | c.-146C>T TERT | C250T | 0,06 | 0,15 | 56,32 | 57,85 | 0 | 0 | 0 | 0 | 0 | 0 |
| HGNMIBC | No | c.746C>G FGFR3 | C228T | 44,63 | 29,96 | 0,11 | 0 | 0 | 0 | 0 | 0,06 | 0,05 | 0 |
| LGNMIBC | No | negative | negative | 0,14 | 0,04 | 0 | 0,05 | 0 | 0 | 0,04 | 0 | 0 | 0 |
| Control | No | negative | negative | 0,07 | 0 | 0 | 0 | 0 | 0 | 0 | 0 | 0 | 0 |
| LGNMIBC | No | negative | C228T | 4,32 | 2,59 | 0,04 | 0 | 0 | 0 | 0 | 0,04 | 0 | 0 |
| MIBC | Yes | c.742C>T FGFR3 (R248C) | C250T + A161C | 0,04 | 0,03 | 4,21 | 3,08 | 0 | 0 | 0 | 0 | 17,59 | 29,14 |
| MIBC | No | negative | negative | 0 | 0,03 | 0,05 | 0,03 | 0 | 0 | 0 | 0 | 0 | 0 |
| Control | No | negative | negative | 0 | 0,02 | 0 | 0 | 0 | 0 | 0 | 0 | 0 | 0 |
| Control | No | c.746C>G FGFR3 (S249C) | C228T | 5,57 | 4,44 | 0,04 | 0,2 | 0 | 0 | 0 | 0,11 | 0 | NA |
| Control | No | negative | negative | 0,04 | 0 | 0 | 0 | 0,04 | NA | 0 | NA | 0,04 | 0 |
| HGNMIBC | No | negative | C228T + C250T | 0,5 | 0,39 | 0,56 | 0,58 | 0,05 | 0,03 | 0 | 0 | 0 | 0 |
| Control | No | negative | negative | 0,04 | 0 | 0 | NA | 0 | NA | 0,04 | NA | 0 | NA |
| Control | No | negative | negative | 0 | 0,03 | 0 | 0,04 | 0 | 0 | 0 | 0 | 0 | 0 |
| HGNMIBC | No | negative | C228T | 28,48 | 55,29 | 0,03 | 0,03 | 0 | 0 | 0 | 0 | 0 | 0 |
| LGNMIBC | No | c.-146C>T TERT | C250T | 0,14 | 0,13 | 6,13 | 3,7 | 0 | 0 | 0 | 0 | 0 | 0 |
| LGNMIBC | No | c.746C>G FGFR3 | C228T | 6,97 | 4,02 | 0,06 | 0 | 0 | 0 | 0 | 0 | 0 | 0 |
| Control | No | negative | negative | 0,04 | 0,02 | 0,09 | 0 | 0 | 0 | 0 | 0 | 0 | 0 |
| Control | No | negative | negative | 0 | 0 | 0 | 0 | 0 | 0,05 | 0 | 0,04 | 0 | 0,18 |
| LGNMIBC | No | negative | A161C | 0,06 | 0,03 | 0,09 | 0 | 0,08 | 0 | 0 | 0 | 1,5 | 0,07 |
| Control | No | negative | negative | 0 | 0,12 | 0,18 | 0 | 0 | 0 | 0,16 | 0 | 0 | NA |
| LGNMIBC | No | c.746C>G FGFR3 | negative | 0 | 0 | 0 | 0 | 0 | 0 | 0,05 | 0 | 0 | 0 |
| HGNMIBC | Yes | negative | C228T | 4,01 | 28,63 | 0,05 | NA | 0 | NA | 0 | NA | 0 | NA |
| HGNMIBC | No | c.-146C>T TERT | C250T | 0,03 | 0,05 | 27,64 | 18,03 | 0,03 | 0 | 0 | 0 | 0 | 0 |
| HGNMIBC | No | c.-124C>T TERT | C228T | 40,17 | 31,85 | 0 | 0 | 0 | 0,04 | 0 | 0 | 0 | 0 |
| Control | No | negative | C228T | 0,59 | 0,27 | 0,05 | 0 | 0 | 0 | 0 | 0 | 0 | 0 |
| MIBC | No | negative | C228T | 21,69 | 32,42 | 0 | 0 | 0 | 0 | 0 | 0 | 0 | 0 |
| LGNMIBC | No | negative | C228T | 12,02 | NA | 0 | NA | 0 | NA | 0,04 | NA | 0 | NA |
| Control | No | negative | negative | 0,04 | 0,02 | 0 | 0,03 | 0 | 0 | 0 | 0 | 0 | NA |
| Control | No | negative | negative | 0 | NA | 0,13 | NA | 0 | NA | 0 | NA | 0 | NA |
| LGNMIBC | Yes | c.-124C>T TERT | C228T | 31,31 | 14,39 | 0 | 0 | 0 | NA | 0 | NA | 0 | 0 |
| LGNMIBC | No | c.746C>G FGFR3 | C228T | 37,32 | 23,07 | 0,04 | 0,04 | 0,06 | 0 | 0 | 0 | 0 | 0 |
| LGNMIBC | No | c.746C>G FGFR3 | C228T | 39,63 | 28,44 | 0,04 | 0 | 0 | 0 | 0,06 | 0 | 0 | 0 |
| HGNMIBC | No | c.-124C>T TERT | C228T | 73,43 | 41,37 | 0,13 | 0 | 0 | 0 | 0 | 0 | 0 | 0 |
| MIBC | No | c.-146C>T TERT & c.742C>T FGFR3 | C250T | 0,08 | 0,05 | 12,81 | 39,17 | 0 | 0 | 0 | 0 | 0 | 0,13 |
| LGNMIBC | No | c.742C>T FGFR3 | negative | 0 | 0,03 | 0,04 | 0 | 0 | 0 | 0 | 0 | 0,05 | 0 |
| Control | No | negative | negative | 0 | 0 | 0,09 | 0 | 0 | 0 | 0 | 0,38 | 0 | NA |
| LGNMIBC | No | negative | C250T | 0 | 0 | 0,9 | 0,62 | 0 | 0 | 0 | 0,14 | 0,04 | 0 |
| LGNMIBC | Yes | negative | negative | 0 | 0,11 | 0,04 | 0,15 | 0 | 0 | 0 | 0,04 | 0 | 0 |
| Control | No | negative | negative | 0 | 0 | 0 | NA | 0 | NA | 0 | NA | 0 | NA |
| MIBC | Yes | negative | C228T | 1,27 | 1,45 | 0 | 0,04 | 0 | 0 | 0 | 0 | 0 | 0 |
| Control | No | negative | negative | 0,04 | 0,08 | 0 | 0 | 0 | NA | 0 | NA | 0 | NA |
| LGNMIBC | No | negative | C228T | 3,07 | 0,65 | 0 | 0 | 0,05 | NA | 0 | NA | 0 | NA |
| Control | No | negative | negative | 0,2 | 0 | 0,04 | 0 | 0 | 0 | 0,04 | 0 | 0 | 0 |
| LGNMIBC | No | negative | C228T | 3,35 | NA | 0,08 | NA | 0 | NA | 0 | NA | 0 | NA |
| Control | No | negative | negative | 0 | 0,09 | 0 | 0 | 0 | NA | 0 | NA | 0 | NA |
| Control | No | negative | negative | 0,14 | 0 | 0,04 | 0 | 0 | 0 | 0 | 0 | 0 | 0 |
| Control | No | negative | negative | 0,06 | 0 | 0 | 0 | 0 | 0 | 0 | 0 | 0 | 0 |
| Control | No | negative | negative | 0,05 | 0 | 0,04 | 0 | 0 | 0 | 0 | 0 | 0 | 0 |
| MIBC | No | c.-124C>T TERT | C228T | 72,3 | 76,56 | 0 | 0 | 0 | 0 | 0 | 0 | 0 | 0 |
| MIBC | No | c.-124C>T TERT | C228T | 31,44 | 42,49 | 0 | 0 | 0 | 0 | 0 | 0,04 | 0 | 0 |
| LGNMIBC | No | c.-146C>T TERT | C250T | 0 | 0,05 | 4,05 | 0,61 | 0 | 0 | 0 | 0,03 | 0 | 0 |
| LGNMIBC | Yes | negative | CC242-243TT | 0,19 | 0,1 | 0,05 | 0 | 0,49 | 0,51 | 0 | 0 | 0 | 0 |
| Control | No | negative | C228T | 0,3 | 0 | 0,04 | 0 | 0,05 | NA | 0 | NA | 0 | NA |
| HGNMIBC | No | negative | negative | 0,05 | 0,04 | 0,05 | 0 | 0 | 0 | 0 | 0 | 0 | 0,05 |
| LGNMIBC | Yes | negative | C228T | 0,66 | 0,31 | 0,12 | 0 | 0 | 0 | 0 | 0,03 | 0 | 0,07 |
| Control | No | negative | negative | 0,11 | NA | 0,08 | NA | 0 | NA | 0 | NA | 0 | NA |
| LGNMIBC | No | negative | C228T | 3,79 | 4,9 | 0 | 0 | 0,08 | 0 | 0 | 0 | 0 | 0 |
| Control | No | negative | negative | 0 | 0 | 0,04 | 0 | 0 | 0 | 0 | 0 | 0 | 0 |
| Control | No | negative | negative | 0,04 | 0 | 0 | 0 | 0 | 0 | 0 | 0 | 0,07 | 0 |
| MIBC | No | c.-124C>T TERT & KRAS G12/13 | C228T | 16,56 | 27,88 | 0 | 0 | 0 | 0 | 0 | 0,06 | 0 | 0,05 |
| HGNMIBC | No | c.-146C>T TERT | C250T | 0 | 0 | 41,16 | 36,47 | 0 | 0 | 0 | 0 | 0 | 0 |
| Control | No | negative | negative | 0 | 0,06 | 0,07 | 0 | 0 | 0 | 0 | 0 | 0 | 0 |
| Control | No | negative | negative | 0 | 0,04 | 0 | 0 | 0 | 0 | 0,04 | 0 | 0 | 0 |
| LGNMIBC | No | c.-146C>T TERT & c.746C>G FGFR3 | C250T | 0 | NA | 12,36 | NA | 0 | NA | 0 | NA | 0 | NA |
| Control | No | negative | negative | 0,05 | 0,06 | 0 | 0 | 0 | 0 | 0 | 0 | 0 | 0 |
| Control | No | negative | negative | 0 | 0 | 0 | 0 | 0,05 | 0 | 0 | 0 | 0 | 0 |
| LGNMIBC | No | negative | C228T | 8,04 | 5,44 | 0 | 0 | 0 | 0 | 0 | 0,11 | 0 | NA |
| MIBC | No | c.-124C>T TERT | C228T | 22,45 | 6,05 | 0 | 0,06 | 0 | 0,09 | 0 | 0 | 0 | 0 |
| MIBC | No | negative | C228T + C250T | 0,28 | 0,18 | 14,84 | 46,27 | 0 | 0 | 0 | 0,07 | 0 | 0 |
| HGNMIBC | No | c.-124C>T TERT & c.746C>G FGFR3 | C228T | 35,97 | 30,46 | 0 | 0 | 0,22 | 0 | 0,07 | 0 | 0 | 0 |
| HGNMIBC | No | negative | negative | 0,04 | 0 | 0,05 | 0 | 0,05 | NA | 0 | NA | 0 | 0 |
| LGNMIBC | Yes | negative | negative | 0 | 0,04 | 0,04 | 0 | 0 | 0 | 0 | 0 | 0 | 0 |
| MIBC | Yes | negative | C228T + C250T | 0,82 | 9,12 | 0,1 | 0,51 | 0,06 | 0 | 0,1 | 0 | 0 | 0 |
| Control | No | negative | negative | 0 | 0,04 | 0 | 0 | 0 | 0 | 0 | 0 | 0 | 0 |
| LGNMIBC | Yes | negative | C250T | 0,05 | 0 | 0,15 | 0,32 | 0 | 0 | 0 | 0 | 0 | 0 |
| Control | No | negative | negative | 0 | NA | 0 | NA | 0,05 | NA | 0 | NA | 0 | NA |
| LGNMIBC | No | negative | negative | 0,04 | 0,03 | 0,06 | 0 | 0 | 0 | 0,04 | 0 | 0 | NA |
| HGNMIBC | No | c.-124C>T TERT | C228T | 29,9 | 34,56 | 0 | 0 | 0 | 0 | 0 | 0 | 0 | 0 |
| HGNMIBC | Yes | negative | C228T | 2,52 | 2,13 | 0 | 0 | 0 | 0 | 0 | 0 | 0 | 0 |
| LGNMIBC | No | c.742C>T FGFR3 | negative | 0,18 | 0 | 0 | 0 | 0 | 0 | 0,06 | 0,15 | 0 | NA |
| HGNMIBC | No | c.-146C>T TERT & c.742C>T FGFR3 | C250T | 0 | 0,04 | 32,07 | 40,67 | 0 | 0 | 0 | 0 | 0 | 0 |
| HGNMIBC | No | c.-124C>T TERT | C228T | 28,11 | NA | 0,07 | NA | 0 | NA | 0 | NA | 0 | NA |
| HGNMIBC | No | c.742C>T FGFR3 | C228T + A161C | 5,77 | 10,42 | 0 | 0,06 | 0 | 0 | 0 | 0 | 0,11 | 0,23 |
| MIBC | No | negative | C250T | 0,27 | 0,05 | 8,21 | 4,06 | 0 | 0 | 0 | 0 | 0 | NA |
| MIBC | No | c.742C>T FGFR3 | C228T | 8,65 | NA | 0,04 | NA | 0 | NA | 0 | NA | 0 | NA |
| LGNMIBC | Yes | negative | negative | 0,29 | 0 | 0 | 0 | 0 | NA | 0 | NA | 0 | NA |
| HGNMIBC | No | negative | C228T | 1,69 | 2,94 | 0,06 | 0 | 0 | 0 | 0 | 0 | 0 | 0 |
| LGNMIBC | No | c.746C>G FGFR3 | C228T | 2,64 | 2,66 | 0,21 | 0 | 0 | 0 | 0 | 0 | 0 | 0 |
| LGNMIBC | Yes | negative | C228T + A161C | 0,51 | 0,32 | 0 | 0 | 0 | 0,04 | 0,05 | 0 | 0,16 | 0,08 |
| Control | No | negative | negative | 0 | 0 | 0 | 0 | 0 | NA | 0 | NA | 0,1 | 0 |
| HGNMIBC | No | negative | negative | 0,04 | 0,04 | 0 | 0 | 0 | 0 | 0 | 0 | 0 | 0 |
| MIBC | Yes | negative | C228T | 7,43 | 13,24 | 0 | 0 | 0 | 0 | 0,04 | 0 | 0 | 0 |
| MIBC | No | c.-146C>T TERT | C228T + C250T | 0,33 | 0,25 | 30,8 | 46,52 | 0 | 0 | 0 | 0 | 0 | 0 |
| LGNMIBC | No | negative | negative | 0,05 | 0 | 0 | 0 | 0 | 0 | 0 | 0 | 0 | 0 |
| MIBC | No | negative | C228T | 11,32 | 21,1 | 0 | 0,04 | 0 | 0 | 0 | 0,18 | 0 | 0,05 |
| HGNMIBC | No | c.-124C>T TERT | C228T | 62,42 | 46,01 | 0 | 0 | 0 | 0 | 0 | 0,16 | 0 | 0 |
| HGNMIBC | Yes | negative | C228T | 7,68 | 26,06 | 0,03 | 0 | 0 | 0 | 0 | 0 | 0 | 0 |
| HGNMIBC | No | c.-124C>T TERT | C228T | 24,8 | 17,65 | 0,07 | 0 | 0 | 0 | 0 | 0 | 0,08 | 0 |
| LGNMIBC | Yes | negative | A161C | 0 | 0 | 0,04 | 0 | 0 | 0 | 0 | 0 | 3,41 | 1,86 |
| MIBC | Yes | c.-124C>T TERT | C228T | 42,07 | 46,45 | 0 | 0,02 | 0 | 0 | 0,06 | 0 | 0 | 0 |
| Control | No | c.742C>T FGFR3 | negative | 0 | 0 | 0,1 | 0 | 0 | 0 | 0 | 0 | 0,07 | 0 |
| Control | No | negative | negative | 0,04 | 0,18 | 0 | 0,05 | 0 | 0 | 0 | 0 | 0 | 0 |
| LGNMIBC | No | negative | negative | 0 | 0,07 | 0,09 | 0 | 0 | 0 | 0 | 0,08 | 0,04 | 0 |
| MIBC | Yes | c.-124C>T TERT | C228T | 19,14 | 45,84 | 0,03 | 0 | 0 | 0 | 0,04 | 0 | 0,03 | 0 |
| LGNMIBC | No | c.-124C>T TERT & c.746C>G FGFR3 | C228T | 40,72 | 31,73 | 0 | 0,06 | 0 | 0 | 0 | 0 | 0 | 0,08 |
| LGNMIBC | No | c.-146C>T TERT & c.742C>T FGFR3 | C250T | 0 | 0,06 | 14,26 | 4,45 | 0 | 0 | 0 | 0 | 0 | 0 |
| HGNMIBC | No | c.-124C>T TERT & c.742C>T FGFR3 | C228T | 34,63 | 34,08 | 0,04 | NA | 0 | NA | 0 | NA | 0,05 | NA |
| LGNMIBC | No | negative | negative | 0,09 | 0 | 0 | 0,05 | 0 | 0 | 0 | 0 | 0 | 0 |
| HGNMIBC | No | c.-146C>T TERT | C250T | 0 | 0 | 29,92 | 32,52 | 0 | 0 | 0 | 0 | 0 | 0 |
| HGNMIBC | No | c.-124C>T TERT | C228T | 41,85 | 23,65 | 0 | 0 | 0 | 0 | 0 | 0 | 0 | 0 |
| HGNMIBC | No | c.-146C>T TERT & c.746C>G FGFR3 | C250T | 0 | 0,1 | 18,88 | 7,39 | 0 | 0 | 0 | 0 | 0 | 0 |
| HGNMIBC | No | c.-146C>T TERT & c.742C>T FGFR3 & c.746C>G FGFR3 | C250T | 0 | 0,04 | 29,72 | 8,08 | 0 | 0 | 0 | 0 | 0 | 0 |
| MIBC | No | c.-124C>T TERT & c.746C>G FGFR3 | C228T | 48,67 | 14,48 | 0 | NA | 0 | NA | 0 | NA | 0 | NA |
| Control | No | negative | negative | 0 | 0,03 | 0 | 0 | 0 | 0 | 0 | 0 | 0 | 0 |
| HGNMIBC | Yes | negative | C250T | 0 | 0 | 0,47 | 0,48 | 0 | 0 | 0 | 0 | 0 | 0 |
| Control | No | negative | negative | 0,15 | 0,04 | 0,06 | 0 | 0,05 | 0 | 0 | 0 | 0,06 | 0 |
| HGNMIBC | Yes | c.-124C>T TERT | C228T | 54,53 | 49,55 | 0,06 | 0 | 0 | 0 | 0 | 0 | 0 | 0 |
| LGNMIBC | No | negative | C250T + C228A | 0 | 0 | 0,22 | 0,13 | 0 | NA | 12,69 | NA | 0,05 | NA |
| Control | No | negative | negative | 0,05 | 0 | 0,13 | 0 | 0 | 0 | 0 | 0 | 0 | 0,07 |
| Control | No | negative | C228T + C250T | 4,18 | 0 | 2,44 | 0,06 | 0 | 0 | 0,14 | 0 | 0 | 0 |
| MIBC | No | negative | C228T | 16,65 | 22,03 | 0 | 0 | 0 | 0 | 0 | 0 | 0 | 0 |
| HGNMIBC | No | negative | C228T | 11,01 | 20,77 | 0,05 | 0,06 | 0 | 0 | 0 | 0 | 0 | 0 |
| Control | No | negative | negative | 0,06 | NA | 0,07 | NA | 0 | NA | 0 | NA | 0 | NA |
| Control | No | negative | negative | 0 | 0,03 | 0,1 | 0 | 0 | 0 | 0 | 0 | 0 | 0,07 |
| Control | No | negative | negative | 0,06 | NA | 0,05 | NA | 0 | NA | 0,05 | NA | 0 | NA |
| Control | No | negative | negative | 0 | 0,05 | 0 | 0 | 0 | 0,08 | 0 | 0 | 0 | 0,14 |
| LGNMIBC | No | negative | C250T | 0 | NA | 11,06 | NA | 0 | NA | 0 | NA | 0 | NA |
| Control | No | c.742C>T FGFR3 | negative | 0,06 | 0 | 0,06 | 0 | 0 | 0 | 0 | 0 | 0 | 0 |
| Control | No | negative | negative | 0,04 | 0,03 | 0 | 0 | 0 | 0 | 0 | 0 | 0 | 0 |
| MIBC | No | negative | C228T | 4,73 | 4,83 | 0,06 | 0 | 0 | 0 | 0 | 0,05 | 0 | 0 |
| Control | No | negative | negative | 0,05 | 0 | 0,22 | 0 | 0 | NA | 0 | NA | 0 | 0 |
| Control | No | negative | C228T | 0,54 | 0,24 | 0 | 0 | 0 | 0 | 0 | 0 | 0 | NA |
| Control | No | negative | negative | 0,1 | NA | 0 | NA | 0 | NA | 0 | NA | 0 | 0 |
| LGNMIBC | No | negative | negative | 0,05 | 0 | 0,06 | 0 | 0 | 0 | 0 | 0 | 0 | 0 |
| Control | No | negative | negative | 0 | 0 | 0 | 0 | 0 | 0 | 0 | 0 | 0 | 0 |
| HGNMIBC | No | c.-124C>T TERT & KRAS G12/13 | C228T | 41,88 | 39,22 | 0 | 0,1 | 0 | 0 | 0 | 0 | 0,05 | 0 |
| HGNMIBC | No | c.-124C>T TERT | C228T | 26,83 | 23,97 | 0 | 0,03 | 0 | 0 | 0 | 0 | 0 | 0 |

**
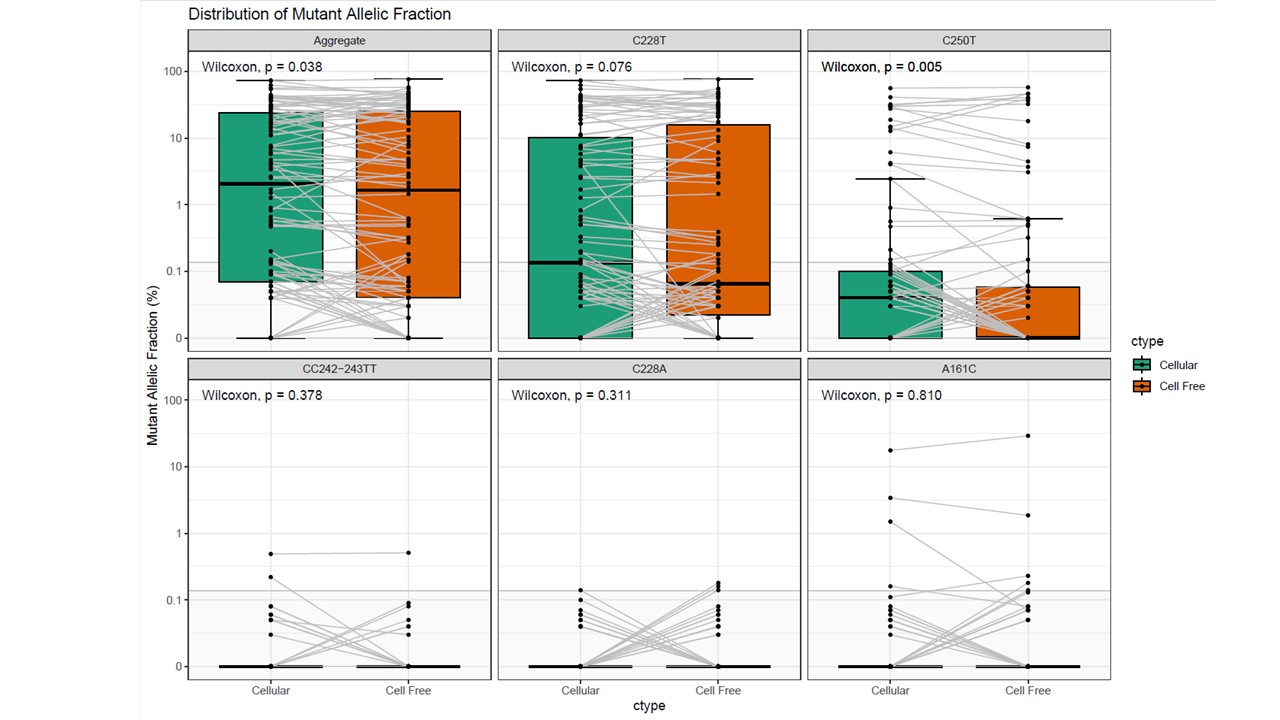
**

**Supplementary Fig. S1.** Comparison of uTERTpm mutant allelic fraction (MAF) in urinary cellDNA (green) and cell-free DNA (red) fractions of 102 paired samples (73 bladder cancer cases and 29 controls). The box plots with whiskers include the median.
